# Collaboration and Satisfaction About Care Decisions in Team questionnaire—Psychometric testing of the Norwegian version, and hospital healthcare personnel perceptions across hospital units

**DOI:** 10.1002/nop2.251

**Published:** 2019-02-20

**Authors:** Oddveig Reiersdal Aaberg, Marie Louise Hall‐Lord, Sissel Iren Eikeland Husebø, Randi Ballangrud

**Affiliations:** ^1^ Faculty of Medicine and Health Sciences, Department of Health Science Norwegian University of Science and Technology Gjøvik Norway; ^2^ Faculty of Health Sciences, Department of Quality and Health Technology University of Stavanger Stavanger Norway; ^3^ Faculty of Health, Science and Technology, Department of Health Sciences Karlstad University Karlstad Sweden; ^4^ Department of Surgery Stavanger University Hospital Stavanger Norway

## Abstract

**Aim:**

To translate “The Collaboration and Satisfaction About Care Decisions in Team” questionnaire (CSACD‐T) into Norwegian and test it for psychometric properties. The further aim was to describe and compare healthcare personnel's collaboration and satisfaction about team decision‐making (TDM) across hospital units.

**Design:**

A cross‐sectional study.

**Methods:**

The questionnaire was translated into Norwegian. A total of 247 healthcare personnel at two hospitals responded to the questionnaire. An explorative factor analysis was performed to test the factor structure of the questionnaire, while a Cronbach's alpha analysis was used to test for internal consistency. A one‐way ANOVA analysis and a Kruskal–Wallis test were applied to test for differences between hospital units.

**Results:**

The results demonstrate that the Norwegian version of the CSACD‐T has promising psychometric properties regarding construct validity and internal consistency. The mean score of the CSACD‐T was significantly higher in the maternity ward group than in the emergency room group.

## 1 INTRODUCTION

The benefits of collaboration and teamwork in health care are well documented (Epstein, 2014; Havyer et al., 2014; Schmutz & Manser, 2013). To achieve a quality of care and patient safety, healthcare personnel need competencies in teamwork (Salas, Cannon‐Bowers, & Johnston, 2014). Team decision‐making (TDM) is a key competency of effective teamwork and important for the results of patient care (Reader, 2017).

## 2 BACKGROUND

Collaboration and teamwork among healthcare personnel include sharing the responsibilities of problem‐solving and decision‐making in formulating and carrying out plans for patient care (O’Daniel & Rosenstein, 2008). TDM refers to the process of reaching a decision among interdependent individuals to achieve a common goal (Bognor, 1997). Healthcare teams may take many forms and range in size. A team is described as a “distinguishable set of two or more people who interact dynamically, interdependently and adaptively towards a common and valued goal, who have each been assigned specific roles or functions to perform and who have a limited life span membership” (Salas, Dickinson, Converce, & Tannenbaum, 1992, p. 4).

Decision‐making and problem‐solving are important parts of everyday practice for hospital healthcare personnel, including physicians, nurses and allied healthcare personnel (Levenson, 2010), with all professions being members of interprofessional teams from time to time (Weinberg, Cooney‐Miner, Perloff, Babington, & Avgar, 2011). Although physicians play an essential role in patient treatment decisions (Farnan, Johnson, Meltzer, Humphrey, & Arora, 2008; Levenson, 2010), nurses and allied healthcare personnel hold patient information that is important in planning, managing and making decisions about patient care and should therefore be involved in the decision‐making process (Marshall, West, & Aitken, 2011). TDM involves both the group which shares information and the team leader who integrates the information and makes a final decision. TDM is important in hospital units, which are often characterized by rapidly changing environments and time pressures (Lipshitz, Klein, Orasanu, & Salas, 2001; Reader, 2017).

Previous research on decision‐making in health care has focused on physicians making decisions about patient treatment (Farnan et al., 2008; Hausmann, Zulian, Battegay, & Zimmerli, 2016), whereas studies on interprofessional collaboration in decision‐making having mostly been about nurse–physician collaboration (DeKeyser Ganz, Engelberg, Torres, & Curtis, 2016; Maxson et al., 2011; Nathanson et al., 2011). Physicians report the most positive perceptions of collaborating in a team; in contrast, nurses are often less satisfied. A limited number of studies have investigated decision‐making in larger hospital teams, beyond nurse–physician teams (Lancaster, Kolakowsky‐Hayner, Kovacich, & Greer‐Williams, 2015; Zwarenstein, Rice, Gotlib‐Conn, Kenaszchuk, & Reeves, 2013). The results of these studies show that care decisions most often take place in isolation by physicians and that the decisions are rarely made collectively. Previous research of multi‐professional TDM across different hospital units has been limited.

Multiple instruments have been developed to measure collaboration in teams (Valentine, Nembhard, & Edmondson, 2015), but not many specific for measuring TDM. The “Collaboration and Satisfaction About Care Decisions in Teams” (CSACD‐T) was designed to measure healthcare personnel's perceptions of collaboration and satisfaction with decision‐making in healthcare teams and is based on the original “Collaboration and Satisfaction about Care Decisions” (CSACD) questionnaire (Baggs, 1994). The original CSACD was developed to measure collaborations between nurses and physicians (Baggs, 1994) and has been used in multiple studies (Klipfel et al., 2011; Maxson et al., 2011; Nathanson et al., 2011; Papathanassoglou et al., 2012) and has also been linked to patient outcomes (Baggs et al., 1999; Boev & Xia, 2015). When the original CSACD was tested for psychometric properties, an EFA was applied only on the first six items of the questionnaire and found the six items to be one factor (Baggs, 1994). When the team version (CSACD‐T) was developed, only minor changes were made in the items with different wordings to capture a broader healthcare team (Fox & Heineman, 2002). The team version of the nine‐item questionnaire (CSACD‐T) has not previously been psychometrically tested.

The theory base of the original CSACD questionnaire was drawn from the work of Thomas (1976), which described a model of collaboration and coordination in complex organizations. According to that model, collaboration is necessary in situations where two or more persons have common interests and the stakes are high. In complex organizations, regarded as dynamic and unpredictable, collaborative solutions provide maximum satisfaction for all parties concerned (Thomas, 1976). Baggs and Schmitt (1988) broadened that model to cover collaborations in decision‐making in health care. Through an extensive literature review, they identified five critical attributes of collaboration: assertiveness, planning, shared decision‐making, open communication and coordination (Baggs, 1994).

An important aspect of TDM in health care is patient participation in decision‐making. The involvement of the patient as a member of the healthcare team is increasingly recognized as a key component of healthcare processes and is advocated as a means to improve patient results and patient safety (Epstein & Gramling, 2013; Longtin et al., 2010; WHO, 2013). An extra item was therefore added to the survey for this study.

We did not find a Norwegian questionnaire that measures healthcare personnel's perception of collaboration in decision‐making in teams. Because CSACD‐T is a brief and simple questionnaire and not profession‐specific, we chose this questionnaire for our study. To the best of our knowledge, no previous studies have investigated TDM among multi‐professional healthcare personnel teams across different hospital units. The aim of the study was to translate “The Collaboration and Satisfaction About Care Decisions in Teams” questionnaire into Norwegian and test it for psychometric properties. The further aim was to describe and compare healthcare personnel's perceptions of collaboration and satisfaction about team decision‐making across hospital units.

## 3 METHODS

### 3.1 Design

This study was designed as a cross‐sectional study. The nine‐item CSACD‐T questionnaire was translated into Norwegian according to a translation‐back‐translation procedure (Brislin, 1970), with further details given in Section 3.4. It was then distributed as a survey to test its psychometric properties.

### 3.2 Setting and sample

In total, 624 healthcare personnel (registered nurses, postgraduate nurses, midwives, occupational therapists, physical therapists, assistant nurses and physicians) were invited to participate in the study. The respondents were from two hospitals in two different hospital trusts in Eastern Norway: 436 from hospital A (110 beds) and 188 from hospital B (167 beds). The healthcare personnel from hospital A (*N* = 436) were from the emergency room (ER), intensive care unit (ICU), operating room (OR)/anaesthesia unit (AN), maternity ward and the medical/surgical (med./surg.) wards, whereas the healthcare personnel from hospital B (*N* = 188) were from medical wards only. All healthcare personnel from the included units were invited to participate in the study. A total of 247 healthcare personnel from the two hospitals responded to the survey.

### 3.3 The CSACD‐T questionnaire

The nine‐item CSACD‐T questionnaire has response options on a Likert scale ranging from 1–7. The first six items measure attributes of collaboration, ranging from 1 (strongly disagree) – 7 (strongly agree). The seventh item measures the level of global collaboration and ranges from 1 (no collaboration) – 7 (complete collaboration). The last two items consider satisfaction with decisions and have response options ranging from 1 (not satisfied) to 7 (very satisfied; Baggs, 1994). The CSACD‐T questionnaire was obtained from its creator, Professor Judith Baggs, and permission was obtained to translate the questionnaire into Norwegian.

Due to the importance of patient participation in care decisions, an extra item (item 10) was developed and added by the research group: “Do patients participate in decision‐making relating to their own care?” The response options ranged from 1 (no participation at all) – 7 (complete participation). This item was not included in the psychometric testing of the questionnaire. The study included the following background data: sex, age, profession, unit type and time employed in the unit.

### 3.4 The translation process

The Brislin Model (Brislin, 1970) was used to translate the English version of the CSACD‐T questionnaire into Norwegian with the following steps:

1. *Forward translation*—Forward translation into the target language (Norwegian) was conducted by three blinded translators: a bilingual professional translator, an American bilingual physician and a Norwegian bilingual academic nurse.
2. *Review*—The research group reviewed the three forward translation versions and compared them with the original version for linguistic congruence and contextual relevance. There were only minor differences among the translators, mostly related to wording. The research group assessed the three versions and agreed on a preliminary translated version. The reconciled Norwegian version was then reviewed by three academic nurses with expert competencies in collaborative care and teamwork in hospitals. Based on their feedback, minor linguistic changes were made.
3. *Back‐translation*—A bilingual professional translator, who was blinded to the original English version, back‐translated the Norwegian version into English.
4. *Compare*—The research group compared the back‐translated version with the original version and found no differences in meaning. Thus, the Norwegian questionnaire was approved for pilot testing.
5. *Pilot testing*—To check for face validity and the understanding of the items in the questionnaire, a pilot test was conducted among multi‐professional healthcare personnel (*N* = 40) from four hospital units in a 180‐bed hospital in another part of the country. The pilot cohort consisted of 19 (47%) registered nurses, 12 (30%) postgraduate nurses, five (13%) physical or occupational therapists and four (10%) physicians. Most of the respondents found the items understandable, well worded, precise and relevant to their profession. Most also indicated that the CSACD‐T was useful for measuring collaboration and satisfaction with decision‐making. Taken together, the results of the pilot study were considered satisfactory and no further changes were made. Lastly, a consensus on the wording of the final Norwegian CSACD‐T version was reached.

### 3.5 Data collection

The survey was distributed as a paper version in November 2015. Two e‐mail‐based reminders were administered, with the assistance of managers, during a data collection period of 3 weeks. Completed surveys were sealed in return envelopes and placed in boxes in the units.

### 3.6 Data analysis

Data analyses were performed with SPSS version 24 (IBM). An explorative factor analysis (EFA) was conducted to test the factor structure of the questionnaire. The aim of the EFA was to test the factor structure of the group of items. In addition to testing the factor structure of the total questionnaire (items 1–9), an EFA was used to analyse items 1–6, due to the intension of comparing our results to those of the original CSACD (Baggs, 1994). Prior to the EFA, we assessed the suitability of our data for factor analysis. This included a correlation matrix for displaying the relationships between the items, as well as correlation coefficients between 0.30–0.70 considered significant (Polit & Beck, 2017; Tabachnick & Fidell, 2013). A Kaiser–Meyer–Olkin (KMO) test was performed to measure sample adequacy. Within the KMO range of 0–1, a value of 0.60 and above was considered suitable for EFA (Pett, Lackey, & Sullivan, 2003; Tabachnick & Fidell, 2013). A principal component analysis (PCA) was chosen for factor extraction. We applied the “eigenvalue rule,” which only allows items of 1.0 or more to be retained for further investigation (Polit & Yang, 2016). A Cronbach's alpha was performed for items 1–9 to check for internal consistency and for items 1–6 to also compare with the original CSACD. A Cronbach's alpha for each item removed was calculated for items 1–9 (Pett et al., 2003).

Descriptive statistics were used to describe the characteristics of the sample and to analyse the results of the CSACD‐T scores. A between‐group one‐way ANOVA, with a Tukey post hoc test, was conducted to compare for differences between unit groups on the total mean score of the healthcare personnel's perceptions of TDM (Polit & Beck, 2017). A Kruskal–Wallis test was conducted to test for differences between groups regarding item 10 (patient participation in decision‐making; Polit & Beck, 2017). A two‐tailed significance level of *p*‐value <0.05 was used for all tests (Polit & Beck, 2017).

### 3.7 Ethical considerations and approvals

“The Norwegian Center for Research Data” approved this study (ref. no. 43295). In addition, the hospital administrations provided approvals. The study was conducted according to the Declaration of Helsinki and ethical guidelines for research (World Medical Association, 2018). The survey included information about the aim of the study, confidentiality and voluntary participation. Completion of the survey was regarded as informed consent.

## 4 RESULTS

### 4.1 Translation and psychometric testing of the CSACD‐T questionnaire

The total of 247 healthcare personnel that responded to the survey represented an overall response rate of 40%. Table 1 shows the distribution of response per healthcare profession.

**Table 1:** Distribution of response per healthcare profession

	Invited *N* = 624	Responded	
		*N* = 247	%
Registered nurse	270	102	38
Postgraduate nurse/Midwife	135	84	62
Assistant nurse	59	27	46
Physiotherapist/Occupational therapist	26	22	85
Physician	110	12	11

Among these individuals, 156 (response rate 36%) were at hospital A and 91 (response rate 48%) at hospital B. Characteristics of the sample are shown in Table 2. Registered nurses constituted most respondents, followed by postgraduate nurses and then midwives. Most of the respondents were female, with 75% of the sample from hospital wards (maternity ward and med./surg. wards). Leaving more than 50% of items blank, two respondents were excluded from further analysis.

**Table 2:** Characteristics of the sample (*N* = 247)

Variable	*N*	%
Sex		
Female	225	91
Male	21	9
Missing	1	
Age		
≤30 years	49	20
31–50 years	119	49
≥51 years	76	31
Missing	3	
Profession		
Registered nurse	102	41
Postgraduate nurse/midwife	84	34
Assistant nurse	27	11
Occupational & physical therapist	22	9
Physician	12	5
Unit type		
Medical & surgical wards	162	65
Maternity ward	24	10
Operation room & Anaesthesia unit	16	6
Intensive care unit	21	9
Emergency room	24	10
Time employed in the unit		
0–5 years	87	35
6–15 years	84	34
≥16 years	75	31
Missing	3	

Inter‐item correlations ranged from 0.45–0.81 for the total questionnaire (items 1–9) and from 0.51–0.81 for items 1–6. All correlations were significant (<0.001). The KMO values were 0.93 for the total questionnaire (items 1–9) and 0.89 for items 1–6. The PCA identified one factor for the total questionnaire (item 1–9), with an eigenvalue of 6.154 on one factor, explaining 68% of the variance and eigenvalues <1.0 on the remaining factor solutions. The PCA on item 1–6 also identified one factor for these items, with an eigenvalue of 4.294 on one factor, explaining 72% of the variance and with eigenvalues <1.0 on the remaining factor solutions (Table 3). PCA factor loadings for the total questionnaire (item 1–9) ranged from 0.72–0.87 and factor loadings for items 1–6 ranged from 0.77–0.89 (Table 4). The Cronbach's alpha was 0.94 for items 1–9 and was not improved when each item was removed (ranged from 0.93–0.94). The Cronbach's alpha value for items 1–6 was 0.92.

**Table 3:** Initial eigenvalues and variance explained—The Collaboration and Satisfaction About Care Decisions in Team questionnaire

Factor component	Eigenvalue item 1–9	% variance explained item 1–9	Eigenvalue item 1–6	% variance explained item 1–6
1	6.154	68. 382	4.294	71. 569
2	0.738	8.205	0.527	8.784
3	0.460	5.116	0.462	7.694
4	0.421	4.678	0.290	4.827
5	0.307	3.412	0.252	4.202
6	0.276	3.070	0.175	2.923
7	0.246	2.731		
8	0.229	2.544		
9	0.167	1.860		

Note

Principal component analysis.

**Table 4:** Factor loadings—The Collaboration and Satisfaction About Care Decisions in Team questionnaire

	Items 1–9	Items 1–6
1. Team members plan together in decision‐making	0.843	0.856
2. Open communication among team members in decision‐making	0.865	0.891
3. Shared responsibilities for decision‐making	0.720	0.774
4. Team members cooperated in decision‐making	0.856	0.884
5. All team members concerns were considered in decision‐making	0.825	0.807
6. Coordination of decision‐making among team members	0.861	0.860
7. Level of collaboration among team members in decision‐making	0.854	
8. How satisfied with the decision‐making process	0.820	
9. How satisfied with the decisions	0.787	

Note

Extraction method: Principal component analysis.

### 4.2 Collaboration in team decision‐making across different hospital units

The entire sample of healthcare personnel's perceptions of TDM showed a total mean score of 5.14 (*SD* = 0.95), as measured by the CSACD‐T (Table 5). Single‐item mean scores for the total sample ranged from 4.82 (*SD* 1.24) (“Coordination of decision‐making among team members”) to 5.35 (*SD* 1.13) (“Shared responsibilities for decision‐making”) (see Table 5). The added item (item 10), which was healthcare personnel's perceptions of “patient participation in decision‐making,” had a mean score of 4.63 (*SD* 1.25) in the total sample (Table 5).

**Table 5:** The healthcare personnel's perceptions of collaboration and satisfaction about care decisions in team

	Total sample *N* = 245		Med/Surg^a^ *N* = 160		MW^b^ *N* = 24		OR/AN^c^ *N* = 16		ICU^d^ *N* = 21		ER^e^ *N* = 24	
	Mean	*SD*	Mean	*SD*	Mean	*SD*	Mean	*SD*	Mean	*SD*	Mean	*SD*
The total CSACD‐T questionnaire (item 1–9)	5.14	0.95	5.20	0.91	5.66	0.88	4.84	0.94	4.89	0.94	4.69	1.06
1. Plan together in decision‐making	5.35	1.13	5.41	1.09	5.82	1.01	4.75	1.18	5.14	1.11	5.04	1.33
2. Open communication in decision‐making	5.32	1.14	5.38	1.14	6.00	0.98	4.94	0.10	4.81	1.12	5.00	1.14
3. Shared responsibilities for decision‐making	5.40	1.30	5.51	1.12	5.55	1.37	5.31	1.45	5.24	1.58	4.75	1.42
4. Team members cooperate in decision‐making	5.29	1.11	5.34	1.07	5.82	0.91	5.13	1.15	5.19	1.03	4.63	1.28
5. All team members` concerns in decision‐making	4.87	1.26	4.96	1.21	5.36	1.09	4.38	1.15	4.62	1.32	4.33	1.55
6. Coordination in decision‐making	4.82	1.24	4.85	1.20	5.64	1.14	4.50	1.10	4.76	1.14	4.17	1.34
7. Level of collaboration in decision‐making	5.07	1.11	5.13	1.04	5.68	1.09	4.88	1.09	4.81	0.98	4.42	1.38
8. How satisfied with the decision‐making process	4.93	1.11	5.00	1.08	5.45	1.01	4.56	1.46	4.43	1.08	4.70	1.02
9. How satisfied with the decisions	5.26	0.96	5.27	0.94	5.64	1.09	5.13	0.96	5.00	1.10	5.22	0.99
10. Patient participation in decision‐making^f^	4.63	1.25	4.81	1.19	5.18	0.96	3.94	1.24	4.33	1.20	3.57	1.20

a Medical/Surgical wards.

b Maternity ward.

c Operating room/Anaesthesia unit.

d Intensive care unit.

e Emergency room.

f The added item for this study.

The results of the one‐way ANOVA revealed statistically significant differences in the total mean score of the CSACD‐T across unit groups (*F*(4, 240) = 4.1, *p *= 0.003). The effect size, calculated using eta squared, was 0.06 (medium effect). Post hoc comparisons, using the Tukey post hoc test, showed a significantly higher score in the maternity group than in the ER group (*p *= 0.001). A Kruskal–Wallis test of item 10 revealed a statistically significant difference between the unit groups (*χ* ^2^(4) = 11.77, *p* = 0.001) with the highest score in the maternity ward group and the lowest score in the ER group.

## 5 DISCUSSION

### 5.1 Translation and psychometric testing of the CSACD‐T questionnaire

The purpose of translating a questionnaire is to obtain an instrument in a new language that is equivalent to the instrument in the original language (Sousa & Rojjanasrirat, 2011). The translation‐back‐translation method used in this study is recommended as a reliable method for translating research instruments (Brislin, 1986; Jones, Lee, Phillips, Zhang, & Jaceldo, 2001). The translation process was thorough; both medical and nursing professionals participated to assure the content and cross‐cultural validity of the questionnaire (Polit & Yang, 2016). No major problems occurred in the translation or back‐translations steps. The respondents in the pilot study provided adequate responses to the items, which suggested that the translated questionnaire was well understood.

The results of the EFA showed one factor for all nine items, and all loadings were above 0.40, which was considered good and the structural validity was thereby supported (Polit & Yang, 2016). Only the first six items were tested and found to be a one‐factor scale in the validation study of the original CSACD (Baggs, 1994). However, when Sapnas, Ward‐Presson, and Monzeglio (2006) conducted a psychometric test to evaluate the original CSACD in diverse hospital unit types, their EFA identified one factor for all nine items of the CSACD questionnaire when tested in diverse hospital unit types, as we found in our study. In any case, since the EFA can only identify clusters of tests that measure the same things, there is no assurance that these “same things” are primary dimensions. Consequently, a factor analysis alone is insufficient to confirm that a factor corresponded directly to the “real” dimension of the construct measured (Polit & Yang, 2016). The interpretation of the results is just as important, a good PCA must make sense (Polit & Yang, 2016). The theory base of the questionnaire included satisfaction with the decisions as an important part of the collaboration in decision‐making (Baggs, 1994). Nonetheless, if the satisfaction part of the questionnaire had included more than two items, the EFA might have resulted in a two‐factor solution.

The CSACD‐T had a Cronbach's alpha value above the desirable 0.80 (Polit & Beck, 2017) and demonstrated a good internal consistency. Result from the previous study of the nine items CSACD showed alpha value over 0.90 (Sapnas et al., 2006). Tavakol and Dennick (2011) argue that the maximum value to be recommended is 0.90, which means that the alpha value of item 1–9 was maybe too high.

Regarding sample size, the recommended sample size for EFA in validation studies is disputed and no consensus exists (Polit & Yang, 2016). Some suggest a minimum of 300, but emphasize that if there is strong correlations and few distinct factors, a smaller sample is adequate (Tabachnick & Fidell, 2013). Others offer guidance on the number of respondents per items, ranging from 5–40 or 50 per item, with the most common recommendation as a minimum of 10 cases per item (Polit & Yang, 2016). The sample size of 247 in the current study was thereby considered satisfactory with 27 number of respondents per item. Multiple types of healthcare personnel from multiple types of hospital units were represented in the sample; hence, a heterogeneous study sample was obtained, as recommended for testing questionnaires (Taber, 2017).

### 5.2 Collaboration in team decision‐making across different hospital units

After translating and testing the psychometric properties of the Norwegian version of the CSACD‐T, we aimed to describe and compare healthcare professional's perceptions of collaboration and satisfaction with TDM across different hospital units. The results showed that the mean CSACD‐T scores were at the same level or slightly higher than those reported in previous studies of nurse–physician collaboration in decision‐making in ICUs (Nathanson et al., 2011; Papathanassoglou et al., 2012) and in paediatric teams (Jankouskas et al., 2007), as measured by the original CSACD. The explanation for why the healthcare personnel from the maternity ward reported a significantly higher score than the healthcare personnel in the ER may be due to having a more team‐based approach to their work (Gregory et al., 2017). Nonetheless, many factors influence TDM such as the amount of work load, time stress and culture (Gregory et al., 2017), as in other types of hospital units.

The lower score in the ER group might be explained by more inefficient teamwork, which may be due to the way clinical work is organized for the sub‐acute patients in ERs in Norway. A nurse or a physician triages the patients and then assigns the patient to a dedicated nurse. By the time the patient's physician arrives, the dedicated nurse has often moved on to attend to the next patient (Krogstad, Lindahl, Saastad, & Hafstad, 2015). The ER is an area of the hospital that is characterized by high complexity, high throughput and high uncertainty and patient care decisions can be affected by the pressures imposed by the high workload and ineffective teamwork (Zavala, Day, Plummer, & Bamford‐Wade, 2018). Team‐based care and TDM are of great importance to ensure quality patient care, in ERs as in other hospital units (Reader, 2017).

The added item “patient participation in decision‐making” had the lowest mean score in the ER group. Although it is well‐known that patients should be included in care decisions, they are still not always included in practice (Williams, Fleming, & Doubleday, 2017). They should be included because they might have valuable information about their own health condition (WHO, 2013). Patient participation may contribute to help healthcare personnel make the right decisions and to minimize decision errors (Zavala et al., 2018). Although healthcare personnel are striving for patient participation in decision‐making to increase the quality of care and patient‐centred outcomes in the ER (Grudzen, Anderson, Carpenter, & Hess, 2016), many patients cannot participate in decisions because of their critical condition (Joseph‐Williams, Elwyn, & Edwards, 2014). In addition, decision‐making in the ER is complex with rapid assessments and decisions to make and includes transferring patients to the next level of care. But for the healthcare team, TDM is possible, as well as being a contribution to safe care, also in the context of the ER (Reader, 2017).

### 5.3 Limitations

Some limitation of the study must be stated. The response rate from physicians was low, which is a common problem in research on healthcare personnel in hospitals (Cunningham et al., 2015). Furthermore, the participants from hospital B were only from medical wards, and except for the med./surg. ward group, unit groups were relatively small. Although this study displayed a relatively low overall response rate, the sample size of 245 respondents was sufficiently large to conduct a factor analysis of the nine‐item questionnaire (Polit & Yang, 2016).

## 6 CONCLUSION

The results of the study demonstrate that the Norwegian version of the CSACD‐T is a questionnaire with promising psychometric properties regarding construct validity and internal consistency. The CSACD‐T questionnaire can be used in assessing collaboration and satisfaction with TDM in hospital teams, in quality improvement, in continuing education endeavours for healthcare personnel and in research. Moreover, the results showed that the levels of collaboration in care decisions in healthcare teams varied across hospital units, with significantly higher scores in the maternity ward group than in the ER group. Further studies are needed with representative samples from diverse hospital units. Additional studies are also needed to confirm our results of the psychometric testing of the questionnaire.

## CONFLICT OF INTEREST

All authors declare no conflict of interest.

## AUTHOR CONTRIBUTION

All the authors made substantial contributions to the conception and design of the study, the analysis and interpretation of data, and the drafting of the manuscript. All the authors critically revised the manuscript for important intellectual content, and all have given their final approval of the version to be published.
